# Exploratory Comparison of Behavior and Welfare-Related Indicators in Captive Lions (*Panthera leo*) Across Three Zoo Enclosures Using Machine-Learning Quantification

**DOI:** 10.3390/ani16193038

**Published:** 2026-09-27

**Authors:** Frej Gammelgård, Silje Marquardsen Lund, Jonas Nielsen, Thea Loumand Faddersbøll, Trine Hammer Jensen, Sussie Pagh, Kirstin Anderson Hansen, Flemming Nielsen, Cino Pertoldi

**Affiliations:** 1 Department of Chemistry and Bioscience, Aalborg University, Frederik Bajers Vej 7H, 9220 Aalborg, Denmarkcp@bio.aau.dk (C.P.); 2 Aalborg Zoo, Mølleparkvej 63, 9000 Aalborg, Denmark; 3 Department of Animal and Veterinary Sciences, Aarhus University, Blichers Allé 20, 8830 Tjele, Denmark; 4 Odense Zoo, Søndre Boulevard 306, 5000 Odense, Denmark; kah@odensezoo.dk; 5 Copenhagen Zoo, Roskildevej 32, 2000 Frederiksberg, Denmark

**Keywords:** computer vision, artificial intelligence, pacing, activity, behavioral diversity

## Abstract

Behavioral monitoring is central to welfare assessment but is difficult to conduct continuously. We used machine-learning-based video analysis to describe lion (*Panthera leo*) behavior across three zoo enclosures differing in available space, structural complexity and social composition. Interpretation of the Aalborg Zoo profile is additionally constrained by a major recent social change, as the enclosure’s adult male was euthanized five days before data collection began. Continuous recordings collected over 10 days were processed with LabGym, filtered for low-confidence predictions and likely static false detections, and summarized as behavioral durations. Because enclosure characteristics, pride size and cub presence varied only between three enclosures, enclosure was treated as the independent comparative unit; repeated hourly and daily observations were used to characterize within-enclosure temporal variation rather than to generate inferential replication. Analyses therefore focused on time budgets, daily and pooled activity, Shannon and Simpson diversity, within- and between-day behavioral instability, social interaction and pacing-like movement. The three enclosures showed distinct behavioral profiles. Diversity was lowest in Aalborg Zoo, social interaction did not increase consistently with pride size, and pacing-like movement was concentrated in Aalborg Zoo. However, the pacing-like measure should be interpreted as a screening indicator, as only 27.7% of manually annotated pacing-like lion-time was retained as pacing-like by the complete pipeline; it therefore cannot be interpreted as an estimate of true pacing prevalence. Activity was highest in Aalborg Zoo, but did not vary consistently with available space or structural complexity across the three enclosures. Automated quantification can support high-resolution exploratory welfare monitoring, but broader inference requires substantially more independently replicated enclosures.

## Simple Summary

Behavioral monitoring is important for assessing welfare in zoo-housed animals, but traditional observations are time-consuming. This study used machine-learning-based video analysis to describe behavior in captive lions housed in three zoo enclosures with different space, structural complexity and social composition. Because these characteristics were represented by only one value per enclosure, the study was treated as an exploratory comparison rather than a statistical test of enclosure effects. Repeated hours and days were used to describe temporal variation within each enclosure, not as independent enclosure replicates. The enclosures showed distinct time budgets, activity profiles and behavioral diversity. Social interaction was highest in the intermediate-sized pride rather than increasing consistently with pride size, and pacing-like movement, used here as an automated screening indicator rather than an estimate of true pacing prevalence, was concentrated in Aalborg Zoo. The results demonstrate how automated video analysis can reveal detailed enclosure-specific behavioral patterns while also illustrating the need for more independently replicated enclosures before environmental or social drivers can be inferred.

## 1. Introduction

Modern zoological organizations, such as the World Association of Zoos and Aquariums (WAZA), the European Association of Zoos and Aquaria (EAZA), and Danish Accredited Zoos and Aquaria (DAZA), play a central role in conservation, research, and public engagement [1,2,3,4,5,6]. In addition, these organizations and their members aim to support biodiversity conservation through breeding programs and education, while maintaining high standards of animal welfare in captive populations [1,2,3,4,5,6]. Welfare is closely linked to an animal’s ability to express species-typical behaviors and cope with environmental and social conditions, making behavioral monitoring a key component of welfare assessment [7,8,9,10]. Continuous welfare assessment, including ensuring good physical and psychological welfare, is embedded in the guidelines and ethical framework for zoos accredited by EAZA; it is therefore important to continuously monitor these animals. [4,8,9,11,12,13]. In this context, large carnivores such as the lion (*Panthera leo*) are of particular interest, as they are especially known to exhibit stereotypic behaviors under suboptimal conditions in captivity, which makes them especially relevant for welfare-oriented behavioral assessment [7,14,15]. Accordingly, lions are widely housed in zoological institutions, where they serve important roles in conservation, education and behavioral research [1,16].

Although certain protected populations have experienced localized increases, the species is undergoing an overall decline across its natural range, largely as a result of habitat loss, human–wildlife conflict, trophy hunting, and unsustainable exploitation [17,18,19,20,21]. Wild populations have undergone substantial range contractions and fragmentation, increasing the importance of ex situ conservation efforts [1,19,20,21]. Lions are unique among felids due to their complex social structure, forming prides that strongly influence behavioral patterns, including activity levels, social interactions, and play behavior [22,23,24]. Although lions spend much of their time resting, active behaviors such as locomotion, exploration, and social play are essential components of their repertoire and are closely linked to environmental and social conditions [25,26,27]. From an ethological perspective, welfare is closely tied to the ability to express such species-typical behaviors [7]. In captive environments, enclosure size, structural complexity, and the availability of stimuli can influence both activity levels and behavioral diversity, while social factors such as pride size and composition affect social interactions [14,15,23,27,28]. Behavioral diversity, defined as the range of behaviors expressed over time, is considered an indicator of positive welfare, whereas reduced activity, limited variation, or the presence of stereotypic behaviors may indicate compromised welfare [7,10,14].

Today, most welfare assessments are conducted through manual observations, either by direct observation or by reviewing video recordings and annotating the specific behaviors performed [29]. This method is highly time-consuming; particularly in long-term behavioral studies, it is difficult to monitor multiple animals simultaneously and if multiple zookeepers or zoologists are annotating the performed behavior, inter-rater reliability may affect the consistency of the data. As a result, supervised computer vision has been adopted to automatically detect and classify animal behaviors from video data [30,31,32,33].

### Aim of This Paper

This study examines how welfare-relevant behavioral patterns differ across three lion enclosures with contrasting physical and social conditions. Lions were categorized into three groups: adult males, cubs of both sexes, and a mixed group consisting of adult females and non-adult individuals of both sexes. Hereafter, the latter group is referred to as the mixed group. Because available space, enclosure complexity, pride size and cub presence each varied at the enclosure level, the study was designed as an exploratory comparative case series rather than a confirmatory test of causal enclosure effects. The following biological predictions were used to structure the comparisons:

1. Enclosures with more available space per lion and greater structural complexity were expected to show a greater proportion of active behavior, including locomotion, marking, feeding and social interaction.
2. Enclosures providing greater behavioral opportunities were expected to show broader behavioral repertoires, expressed as higher Shannon and Simpson diversity. Within-day and between-day instability were additionally quantified to distinguish behavioral diversity from temporal fluctuation.
3. Social interaction was expected to be more frequent in larger prides and in the enclosure containing cubs.
4. Pacing-like movement was expected to differ descriptively among enclosures.

## 2. Materials and Methods

### 2.1. Video Collection and Processing

Video recordings were collected between 22 and 31 December 2025, using eight mini PTZ Wi-Fi IP outdoor surveillance cameras (Camwon PTZW-IP5S500F (manufactured in Guangzhou, Guangdong Province, China)). Although the cameras were PTZ-capable, camera orientation and zoom were kept fixed during data collection. Detailed camera placement, availability, recording stability, blind spots and camera-specific prediction coverage are reported in the accompanying methodological study [34]. The cameras were configured for continuous recording, although camera availability differed among institutions, resulting in approximately 1830 h of video footage. The recordings were all processed using a complex LabGym model, which categorizes lion behaviors on a 1/10 s level. All camera and model specifications, including camera placement, video preprocessing and post-processing, model development, internal validation, and behavior-specific applied validation against temporally held-out manually annotated video are described in detail in the accompanying methodological study: “A Standardized Methodological Framework for Model Development in LabGym: A Case Study in Lions” [34]. Internal categorizer performance and summary results from the temporally held-out applied validation are additionally provided in Appendix B, Table A1, Table A2 and Table A3.

### 2.2. Lion Enclosures

To evaluate how enclosure characteristics, social structure, and zoo care and management practices may influence lion behavior and welfare, background information was collected from each zoological institution; Aalborg Zoo (Aalborg, Denmark), Odense Zoo (Odense, Denmark) and Zoo Copenhagen (Copenhagen, Denmark), respectively referred to as AAL, ODE and KBH. This section provides an overview of each zoo, including enclosure layout and size, pride composition and daily management routines. The information was obtained directly from staff at each zoological institution through structured questionnaires and follow-up correspondence. We assessed the structural complexity of each enclosure based on the following features: vegetation cover, elevated platforms, caves or shelters, vantage point hills, water features, indoor–outdoor access, outdoor visual barriers, marking posts or trees, movable objects, static enrichment and feeding apparatus. These features were considered collectively as a descriptive assessment of the structural opportunities available within each enclosure. No individual feature was formally weighted, and the resulting complexity score was not intended as a standardized or continuous ecological metric. Instead, the three enclosures were ranked relative to one another from 1 (lowest structural complexity) to 3 (highest structural complexity) based on the overall presence and variety of these features. Subspecies were excluded from further analyses due to recent revisions in lion taxonomy (Appendix A), potential hybridization within captive populations, and uncertainty regarding the consistency of classifications among zoological institutions.

#### 2.2.1. Aalborg Zoo Lion Enclosure

Relevant information about the AAL enclosure can be found in Table 1.

The Aalborg Zoo pride had experienced a major recent social change immediately before the study: the only adult male was euthanized on 17 December 2025, five days before data collection began. Because this event may have influenced the behavior of the remaining females during the observation period, AAL should be considered a case observed shortly after a substantial change in pride composition rather than a socially stable baseline. The two females (mixed group), a mother and her adult daughter, were 12 and 6 years old, respectively, and originated from Zoo Magdeburg and Edinburgh Zoo.

#### 2.2.2. Zoo Copenhagen Lion Enclosure

Relevant information about the KBH enclosure can be found in Table 2.

The Copenhagen Zoo pride consisted of one adult male and six individuals belonging to the mixed group. The adult male was 15 years old and was born at Givskud Zoo, arriving at Zoo Copenhagen in March 2014. The mixed group consisted of two adult females and four one-year-old individuals originating from the same litter. The adult females were sisters, both 13 years old, and were born and raised at Zoo Copenhagen.

#### 2.2.3. Odense Zoo Lion Enclosure 

Relevant information about the ODE enclosure can be found in Table 3.

The Odense Zoo pride consisted of one adult male, five individuals belonging to the mixed group, and three cubs. All adult individuals were approximately 4.5 years old at the time of the study. The adult male and one of the adult females were born at Odense Zoo, while the two remaining adult females were transferred from Zoo Copenhagen in August 2022. The two individuals in the mixed group were approximately 1.5 years old, whereas the three cubs were approximately four months old at the time of the study.

### 2.3. Ethogram

Lion behaviors were classified using a study-specific ethogram (Table 4) adapted from Stanton et al. (2015) [35]. The ethogram was designed to capture behaviors relevant to activity, social interaction and welfare assessment in captive lions. The final behavioral categories used for quantification included feeding, inactive, interacting, locomotion, marking, self-grooming and vocalization. The behavioral categories were operational categories developed for automated classification rather than measures of behavioral valence. In particular, the interacting category quantified the occurrence of direct social interaction but did not distinguish affiliative, reproductive or agonistic interactions and should therefore not be interpreted as a measure of positive or negative social welfare. Similarly, the inactive category was an operational low-movement category rather than a distinction among different stationary behavioral states. The model did not distinguish vigilance or observation from resting or sleeping; these states were grouped as inactive when the lion remained stationary or nearly stationary and did not meet the criteria for another behavioral category. Pacing-like movement was treated separately from the main LabGym behavior categories because it represents a repeated movement pattern rather than a single frame-level behavior. It was therefore assessed using tracking-based post-processing rather than direct behavior classification.

### 2.4. Data Analysis

Behavioral analyses were conducted in R (version 4.4.2) [36] using cleaned output from LabGym. Frame-level behavior predictions were converted into behavior durations, measured in seconds, and summarized by enclosure, date, hour, animal class and behavior category. Low-confidence predictions were excluded using a confidence threshold of 0.60. This threshold was established during model development, before evaluation of the temporally held-out validation footage, as a balance between retaining potentially correct predictions and excluding low-confidence classifications. Consequently, all behavioral proportions reported below are conditional on retained classified behavior time after confidence and post-processing filters, rather than on total video-recording duration or all observable lion-time. Because some night-time recordings contained static false detections, an additional movement-based filter was applied to recordings from the defined night period. Between 17:00 and 08:00, likely static false detections were evaluated in 300 s windows. A window was removed when the track was detected for at least 10 s, at least 80% of frame-to-frame centroid displacements were <5 pixels, and the median frame-to-frame displacement was ≤1 pixel. The static-filtering procedure was applied before confidence filtering and behavioral aggregation.

Stable individual identity was not retained in the downstream analyses. Occlusion or overlap between animals could therefore result in temporary detection loss or fragmentation of tracking sequences rather than reconstruction of individual identity through the overlap. Behavioral outputs were consequently summarized at the animal-class level. For pacing analysis, short tracking gaps (≤1 s) could be linearly interpolated, whereas gaps exceeding 2 s resulted in tracklet separation. Usable animal-tracking time was calculated from the elapsed duration of analyzed tracklets after this tracking-gap processing, rather than from raw video duration or by summing overlapping pacing-detection windows. Each simultaneously tracked animal contributed separately to this denominator, so usable animal-tracking time represents accumulated tracked animal-time rather than camera-recording time. The complete tracking and post-processing procedure is described in [34]. Prolonged likely static false detections during night-time recordings were addressed using the movement-based filtering procedure described above.

The statistical unit was defined according to the level at which each predictor varied. Available space, complexity, pride size and cub presence each had one value per enclosure; consequently, the independent comparative sample for these variables consisted of three enclosures. Hourly and daily observations were repeated measurements nested within an enclosure and were used to quantify temporal variation and improve the description of each enclosure, but they were not treated as additional independent replicates for between-enclosure inference [37,38]. Results are presented as enclosure-level proportions together with daily distributions, medians, interquartile ranges (IQRs), ranges and descriptive contrasts.

#### 2.4.1. Time Budgets and Boxplots

To describe the overall behavioral composition of the dataset, time budgets were calculated for each enclosure. For each behavior category, total observed duration was divided by total classified behavior time within the enclosure. Time budgets were calculated both across the full study period and separately for each day. Daily boxplots and individual daily points were used to show temporal variation within enclosures. These daily values were interpreted as repeated observations of the same enclosure rather than independent enclosure replicates.

#### 2.4.2. Activity and Enclosure Characteristics

Behaviors were grouped into active and non-active categories. Active behaviors included locomotion, marking, vocalization, interacting and feeding. Active duration and total observed duration were first summarized by enclosure-hour and then by enclosure-day. For each enclosure, a pooled activity proportion was calculated as total active duration divided by total observed duration across the study period. Daily activity proportions were summarized using the median, IQR and range to show within-enclosure temporal variation. The three pooled enclosure estimates were displayed alongside available space (m^2^ per lion) and the assigned complexity score. Because only three independent enclosure-level observations were available, no regression line or significance test was fitted; the ordering of the observations was described only as monotonic or non-monotonic. A pattern was considered directionally consistent with the expectation when the rank ordering of activity followed the rank ordering of the enclosure characteristic across all three enclosures. If the ordering differed for one or more enclosures, the pattern was described as non-monotonic.

#### 2.4.3. Behavioral Diversity and Behavioral Instability

Behavioral diversity was calculated from daily enclosure-level behavior totals using Shannon and Simpson indices [10,39]. Behavior categories not observed during an otherwise usable hour or day were represented by zero rather than being omitted. Shannon diversity was used because it is relatively sensitive to rare behaviors, whereas Simpson diversity gives greater weight to dominant behaviors [39]. The diversity estimates should additionally be interpreted in relation to classification performance. Because Shannon diversity is comparatively sensitive to rare behavioral categories, errors in detecting or classifying infrequent behaviors may disproportionately affect this index. The applied validation of the analytical pipeline showed incomplete prediction coverage and substantial variation in performance among behavioral categories [34]. Rare behaviors may therefore be underestimated when observations are missed or assigned to more common categories, while false-positive classifications may conversely increase their apparent contribution. Fine-scale behavioral time budgets and Shannon diversity should consequently be interpreted as descriptive estimates subject to classification uncertainty, rather than precise measures of the underlying behavioral repertoire.

Two complementary instability measures were calculated. Within-day instability quantified fluctuation across hours: for each enclosure-day and behavior, the IQR of hourly behavior proportions was calculated, and the mean of these behavior-specific IQRs was used as the daily within-day instability score [40]. Between-day instability quantified variation across recording days: for each enclosure and behavior, the IQR of daily behavior proportions was calculated, and the mean of these behavior-specific IQRs was used as the enclosure-level between-day instability score. Thus, a larger within-day value indicates greater hour-to-hour change in the behavioral time budget, whereas a larger between-day value indicates greater variation in behavioral allocation among recording days. Daily Shannon, Simpson and within-day instability values were summarized within each enclosure using descriptive statistics. No between-enclosure rank tests were used.

#### 2.4.4. Social Behavior, Pride Size and Cub Presence

Social behavior was defined from the interacting category. For each enclosure-hour and enclosure-day, social-interaction duration was divided by total observed behavior duration. A pooled social-interaction proportion was then calculated for each enclosure, with daily median, IQR and range used to describe temporal variation. These three enclosure-level estimates were displayed alongside pride size and cub presence. No regression or significance test was performed because pride size was confounded with enclosure identity and cubs were present in only one enclosure.

#### 2.4.5. Pacing-like Behavior

Pacing-like movement was analyzed separately from the LabGym behavior categories because it is defined by repeated movement patterns rather than a single posture or frame-level label. Tracking data were used to identify sustained back-and-forth movement consistent with pacing. Pacing-like bouts were summarized by enclosure and day as total pacing duration, number of bouts and pacing duration relative to usable animal-tracking time. Because the detection windows overlap, usable tracking time was calculated from the time span of each analyzed tracklet rather than by summing window durations. Enclosure-level pacing was summarized descriptively using pooled pacing proportion, the proportion of days on which pacing-like movement was detected, and the median and IQR of daily pacing proportions. No between-enclosure significance test was performed. The detection algorithm is described in detail in the companion methodological article: “A Standardized Methodological Framework for Model Development in LabGym: A Case Study in Lions” [34].

#### 2.4.6. Spatial Analysis of Enclosure Use

To complement the behavioral analyses, spatial heatmaps were generated from centroid tracking derived from the LabGym detector model. For each frame with a detected lion, the centroid of the bounding polygon was used and aggregated across the recording period. For each heatmap, up to 300,000 centroids were randomly selected from the full dataset to standardize visualization. Heatmaps were used descriptively to compare spatial-use patterns among camera views and enclosures and to provide context for activity and pacing-like movement.

## 3. Results

### 3.1. Time Budgets

To provide an overview of the behavioral composition, time budgets were calculated for each enclosure, first as an overall time budget across the full study period (Figure 1) and secondly as daily time budgets (Figure 2). After confidence and post-processing filters, the total retained classified behavior time was 580,998.7 s (161.4 h) in AAL, 553,514.9 s (153.8 h) in KBH and 571,714.2 s (158.8 h) in ODE. Time-budget proportions were calculated relative to these enclosure-specific totals. Among night-time tracking windows containing at least 10 s of detected tracking and therefore eligible for the static filter, 8714 of 9554 windows (91.2%) in AAL, 4641 of 4943 (93.9%) in KBH, and 10,510 of 11,566 (90.9%) in ODE met the static-movement criteria and were removed. The overall time budget showed that locomotion was the most frequently observed behavior in AAL, accounting for 44.3% of observed behavior time. In contrast, inactive behavior was the most frequently observed behavior in KBH and ODE, accounting for 41.9% and 45.3% of observed behavior time, respectively.

Interacting behavior was most prevalent in KBH, where it accounted for 21.8% of observed behavior time, followed by AAL at 17.3% and ODE at 11.2%. Feeding and self-grooming were most prevalent in ODE, accounting for 5.9% and 7.6% of observed behavior time, respectively. The same behaviors were less frequent in KBH, where feeding accounted for 2.2% and self-grooming for 3.6%, and least frequent in AAL, where feeding accounted for 0.6% and self-grooming for 2.8%. Marking and vocalization were rare in all enclosures. Because fine-scale estimates of rare behaviors are particularly sensitive to classification uncertainty, these categories were not interpreted individually.

The daily time budgets showed broadly similar patterns to the overall time budgets. The daily variation in behavior-specific time budgets is presented as boxplots in Appendix C.

### 3.2. Spatial Use of Enclosure

Spatial heatmaps showed differences in the distribution of detected lion centroid positions across the three institutions (Figure 3, Figure 4 and Figure 5). In Aalborg Zoo, detections were concentrated in a limited number of areas, with repeated high-density zones along particular paths. In contrast, Odense Zoo showed a more distributed pattern of detections, with multiple areas of moderate to high density across the enclosure, particularly around the heated rock and permanent enrichment. Zoo Copenhagen showed higher densities of detected positions along enclosure edges and elevated areas, with fewer detections in the central parts of the enclosure.

Overall, the heatmaps indicate differences in the spatial distribution of detected lion positions among the three enclosures. These patterns are presented descriptively and should not be interpreted as validated quantitative estimates of enclosure occupancy or preference.

### 3.3. Activity in Relation to Enclosure Space and Complexity

The three enclosures differed substantially in available space per lion and complexity score. AAL provided 350.0 m^2^ per lion and had a complexity score of 2, KBH provided 100.4 m^2^ per lion with a complexity score of 1, and ODE provided 138.9 m^2^ per lion with a complexity score of 3. Pooled activity was highest in AAL (63.1%), intermediate in KBH (54.8%), and lowest in ODE (47.1%). The same ordering was observed for median daily activity, which was 64.6% in AAL, 55.7% in KBH and 46.6% in ODE. Daily variability was similar among enclosures, with IQRs of 9.0, 8.0 and 9.1 percentage points, respectively. The activity ranking therefore did not correspond consistently to the ranking in available space. AAL had both the greatest available space and the highest activity, but ODE provided more space per lion than KBH (138.9 vs. 100.4 m^2^) while showing lower activity (47.1% vs. 54.8%). Activity also showed no monotonic increase with complexity: activity increased from complexity score 1 in KBH to score 2 in AAL, but was lowest in ODE, despite ODE having the highest complexity score of 3. These patterns are interpreted descriptively because space and complexity varied only among the three enclosures.

### 3.4. Behavioral Diversity and Behavioral Instability in Relation to Environmental Opportunity

Behavioral diversity differed descriptively among the three enclosures. Mean Shannon diversity was lowest in AAL (1.189 ± 0.053), intermediate in KBH (1.294 ± 0.061), and highest in ODE (1.359 ± 0.085). Mean Simpson diversity showed a somewhat different pattern, being lowest in AAL (0.642 ± 0.033) but similar in KBH (0.682 ± 0.024) and ODE (0.677 ± 0.039). Thus, AAL consistently showed the lowest behavioral diversity, whereas the distinction between KBH and ODE depended on the diversity index used. Within-day behavioral variability was also greatest in AAL. Median within-day variability was 0.154 (IQR = 0.037) in AAL, 0.126 (IQR = 0.019) in KBH, and 0.110 (IQR = 0.023) in ODE. Hour-to-hour behavioral allocation was therefore most variable in AAL and least variable in ODE. Between-day variability showed a similar but not identical ordering. The enclosure-level between-day variability score was 0.041 in AAL, 0.034 in ODE and 0.029 in KBH. AAL therefore combined the lowest behavioral diversity with the greatest temporal variability, whereas ODE combined the highest Shannon diversity with the lowest within-day variability. KBH showed intermediate Shannon diversity but the lowest between-day variability.

### 3.5. Social Interaction in Relation to Pride Size and Cubs

The enclosures differed in pride size and cub number: AAL housed 2 lions and no cubs, KBH housed 7 lions and no cubs, and ODE housed 9 lions including 3 cubs. Social interaction accounted for 17.3% of observed behavior time in AAL, 21.8% in KBH and 11.2% in ODE. Thus, the descriptive pattern was non-monotonic with pride size: the highest social-interaction proportion occurred in the intermediate-sized pride rather than the largest pride. The only enclosure containing cubs, ODE, did not show the highest social-interaction proportion. Cub presence therefore cannot be separated from the other characteristics unique to ODE.

### 3.6. Pacing-like Locomotion

Pacing-like movement was concentrated in AAL. Across the 10 analyzed days, 11,350 s of pacing-like locomotion were detected from 4,311,785 s of usable animal-tracking time, corresponding to a pooled pacing-like proportion of 0.263%. Pacing-like movement was detected on four of the ten analyzed days, with a mean of 1135 s and 20.7 detected bouts per day. KBH showed substantially less pacing-like movement, with 60 s detected from 7,274,814.7 s of usable animal-tracking time, corresponding to a pooled proportion of 0.000825%. This represented an average of 6 s and 0.2 bouts per day, with pacing-like movement detected on two of ten days. ODE showed similarly little pacing-like movement, with 35 s detected from 5,416,252.2 s of usable animal-tracking time, corresponding to a pooled proportion of 0.000646%. This represented an average of 3.5 s and 0.1 bouts per day, with pacing-like movement detected on one of ten days. The concentration of pacing-like movement in AAL occurred in the context of the euthanasia of the pride’s only adult male five days before data collection began.

## 4. Discussion

### 4.1. General Interpretation and Study Limitations

This study used machine-learning-based behavioral quantification to compare three zoo-housed lion groups across continuous recordings. The video pipeline generated many hourly and daily observations, but available space, complexity, pride size and cub presence each remained properties of only three enclosures. Treating the repeated temporal observations as independent replicates of those enclosure-level variables would inflate the apparent sample size and could create pseudoreplicated inference [37,38]. The analysis therefore uses hours and days to characterize each enclosure in detail while restricting conclusions about environmental and social factors to descriptive patterns. A further important limitation concerns the recent social history of AAL. The pride’s only adult male was euthanized five days before data collection began, meaning that the remaining females were observed shortly after a substantial change in group composition. This event provides a plausible contributing explanation for the broader AAL profile, including its comparatively high activity, low behavioral diversity, greater temporal instability and concentration of pacing-like movement. However, because no behavioral baseline from before the event was available, its contribution cannot be separated from other AAL-specific characteristics, including enclosure design, husbandry and individual history.

The study can describe what was observed in AAL, KBH and ODE during the recording period, including the magnitude and temporal consistency of behavioral differences. It cannot determine whether space, complexity, pride size or cub presence caused those differences, because each factor is confounded with other characteristics of the same enclosure, group and husbandry regime.

### 4.2. Hypothesis 1: Activity in Relation to Enclosure Space and Complexity

The prediction that greater available space and structural complexity would be associated with a higher proportion of active behavior received only limited descriptive support. AAL, which provided substantially more space per lion than the other enclosures, also showed the highest pooled activity proportion. However, the pattern was not consistent across all three enclosures: ODE provided more space per lion than KBH but showed lower activity. Available space therefore did not show a simple monotonic relationship with activity. Similarly, activity did not increase consistently with the ordinal complexity score. AAL, with an intermediate complexity score, showed the highest activity, whereas ODE had the highest complexity score but the lowest activity. These patterns do not rule out a role of enclosure space or environmental complexity, but they demonstrate that the observed differences cannot readily be attributed to either characteristic in isolation. It should also be noted that the recent euthanization of the only male lion in the AAL enclosure may also be a contributing factor to the higher activity levels. From a welfare perspective, opportunities for activity and environmental engagement remain relevant because locomotion, exploration and interaction with the environment form part of species-typical behavioral expression [9,12,16,28]. At the same time, lions naturally spend a substantial proportion of their time resting, and a high proportion of inactivity should therefore not automatically be interpreted as impaired welfare [22,41]. Rather than activity alone, the opportunity to express an appropriate range of active and inactive behaviors may be more informative [15,28,42]. The absence of a straightforward relationship with complexity may partly reflect how complexity was represented in the present study. The ordinal complexity score summarized multiple enclosure features into a single ranking and could not capture how individual features were used by the animals. Moreover, complexity was inseparable from other differences in management routines, pride composition and feeding schedules. Consequently, the present results should not be interpreted as evidence that structural complexity has no effect on behavior, but rather that its relationship with activity could not be isolated using three contrasting enclosure cases.

The spatial heatmaps (Figure 3, Figure 4 and Figure 5) provided an additional perspective by visualizing how lions used the available enclosure space. Clear differences in spatial-use patterns were apparent among the three zoological institutions, with some enclosures showing concentrated use of particular areas and others showing more distributed use. Concentrated spatial use may reflect preferred resting or activity locations, habitual routes or restricted use of particular areas, whereas broader spatial distributions may indicate use of a greater proportion of the enclosure. However, these patterns should also be interpreted in relation to tracking quality. Some highly concentrated areas may reflect repeated false detections, particularly around rocks or other background objects that could be misidentified as lions during low-light recordings. The heatmaps should therefore be considered as complementary descriptive information rather than direct measures of enclosure quality.

### 4.3. Hypothesis 2: Behavioral Diversity and Behavioral Instability

Behavioral diversity also differed descriptively among the three enclosures, although the pattern did not correspond straightforwardly to the ordinal complexity ranking. AAL showed the lowest mean Shannon and Simpson diversity, while ODE showed the highest Shannon diversity. Simpson diversity was similar in KBH and ODE. Thus, the results indicate a comparatively narrower behavioral distribution in AAL, whereas KBH and ODE showed broader behavioral profiles. However, because KBH had the lowest complexity score while AAL had an intermediate score, behavioral diversity did not increase monotonically with the assigned measure of structural complexity. The prediction that greater behavioral opportunity would be associated with greater behavioral diversity therefore received some descriptive support, particularly for ODE, but was not consistently supported across all three enclosures.

The distinction between Shannon and Simpson diversity remains biologically relevant. Shannon diversity is comparatively sensitive to rarer behaviors, whereas Simpson diversity is influenced more strongly by dominant behaviors. The higher Shannon diversity in ODE may therefore indicate that behavioral time was distributed across a broader range of behaviors, including less frequently expressed categories, whereas the similarity in Simpson diversity between ODE and KBH suggests that their dominant behavioral categories were distributed more similarly. Behavioral diversity may consequently reveal aspects of behavioral organization that are not captured by total activity alone [10,39]. However, greater behavioral diversity should not automatically be equated with better welfare without considering which behaviors contribute to the index. A diverse repertoire dominated by species-typical behaviors has a different welfare interpretation from one that includes substantial abnormal or stress-related behavior [7,10,43]. Behavioral instability provided a complementary measure of temporal organization. Whereas Shannon and Simpson diversity describe how behavioral time is distributed among categories, instability describes how strongly this distribution changes over time [40]. Within-day instability was descriptively highest in AAL, intermediate in KBH and lowest in ODE. Between-day instability showed a slightly different ordering: AAL again showed the greatest instability, whereas KBH showed the lowest and ODE was intermediate. These patterns demonstrate that behavioral diversity and temporal instability captured different features of the behavioral profiles.

In particular, AAL combined the lowest behavioral diversity with the greatest temporal instability at both temporal scales. Its behavioral repertoire was therefore comparatively less diverse overall but more variable in its allocation across hours and days. ODE, in contrast, combined the highest Shannon diversity with the lowest within-day instability, indicating that a comparatively broad behavioral repertoire could occur alongside relatively stable hourly behavioral allocation. KBH showed intermediate Shannon diversity but the lowest between-day instability. These patterns illustrate why behavioral diversity and instability should not be treated as interchangeable indicators. High diversity may indicate expression of a broader behavioral repertoire, whereas temporal instability describes how consistently that repertoire is organized through time.

Instability should nevertheless be interpreted cautiously because IQR-based measures can be influenced by the number and distribution of usable observations, animal visibility and model detection quality. It should therefore be considered as a descriptive measure of temporal fluctuation rather than a direct welfare score. The repeated daily observations in the present study were used to characterize this variation within each enclosure and not as independent evidence of enclosure-level effects.

Classification performance represents an additional limitation when interpreting behavioral diversity and time budgets. Because Shannon diversity is comparatively sensitive to rare behaviors, errors in detecting or classifying infrequently expressed behaviors may disproportionately influence the index. Rare behaviors may be underestimated when observations are missed or assigned to more common categories, whereas false-positive classifications may conversely increase their apparent contribution. The applied validation of the analysis pipeline demonstrated incomplete prediction coverage and variation in classification performance among behavioral categories [34]. Accordingly, fine-scale time budgets and diversity estimates should be interpreted as descriptive measures that remain subject to classification uncertainty.

Overall, the prediction concerning behavioral diversity received partial descriptive support. The three enclosures showed distinct diversity and instability profiles, but these differences did not follow a simple gradient in structural complexity. More importantly, the results demonstrate that overall behavioral diversity and temporal stability provide complementary information that would be obscured if behavioral variation were represented by a single metric.

### 4.4. Hypothesis 3: Social Interaction in Relation to Pride Size and Cub Presence

The prediction that social interaction would increase with pride size and cub presence was not supported by the descriptive pattern. Social interaction was highest in KBH, which housed the intermediate-sized pride, followed by AAL, which housed the smallest pride, and was lowest in ODE, which housed the largest pride. Thus, social interaction did not increase monotonically with pride size. Likewise, ODE was the only enclosure containing cubs but showed the lowest proportion of social interaction. These observations should be interpreted with particular caution. Cubs were present in only one enclosure, meaning that cub presence was completely confounded with the other characteristics specific to ODE. Pride size likewise varied only between the three enclosures and could not be separated from enclosure design, husbandry routines, age and sex composition or group history. The results therefore show that these two simple group-level characteristics were insufficient to explain the observed differences among the three prides. The pattern may also partly reflect how social behavior was quantified. In the present study, social behavior was represented by the interaction category, which included a range of direct interactive behaviors. Lions may nevertheless exhibit socially relevant behavior that does not involve clearly observable interaction. For example, individuals in larger prides may rest in proximity or coordinate their spatial behavior without displaying movements that are classified as interaction. Furthermore, social relationships in lions are influenced not only by group size, but also by kinship, age, sex composition, reproductive state, pride stability and recent social events [22,23,27]. These factors may therefore be more informative than pride size and cub presence alone when interpreting social behavior. Accordingly, the interaction proportions reported here represent the frequency of detected direct interaction and cannot determine whether the social exchanges were predominantly affiliative, reproductive or agonistic.

### 4.5. Hypothesis 4: Quantification of Pacing-like Movement

Pacing-like movement showed one of the clearest descriptive differences among the three enclosures. Pacing-like movement was concentrated primarily in AAL, whereas only very limited amounts were detected in KBH and ODE. This is consistent with the prediction that pacing-like movement would differ descriptively among enclosures. However, because the study included only three independent enclosure cases, the observed pattern cannot identify which enclosure, social or management characteristics contributed to the difference.

One potentially important contextual factor is the recent social history of the AAL pride. The pride’s only adult male was euthanized five days before data collection began, meaning that the remaining females were observed shortly after a substantial change in group composition. This social perturbation may have contributed to the concentration of pacing-like movement observed in AAL. However, because no behavioral baseline from before the event was available and the study was not designed to evaluate its consequences, its contribution cannot be separated from other AAL-specific factors such as enclosure characteristics, husbandry and individual history.

Pacing estimates also depend strongly on tracking quality, camera angle and the ability of the post-processing algorithm to distinguish sustained repetitive back-and-forth movement from other forms of locomotion. Automated identification of pacing-like movement may consequently be affected by fragmented tracks, false detections or repeated route-based movement that is not stereotypic. Repeated use of particular areas visible in the spatial heatmaps could, for example, represent habitual movement routes that resemble pacing according to movement-based criteria. Stereotypic behavior is commonly considered potentially relevant to welfare assessment, but its interpretation depends strongly on behavioral and environmental context [7,13,14]. The pacing-like detection procedure has also been evaluated against manually annotated video as part of the temporally held-out applied validation [34]. Prediction coverage during manually annotated pacing-like periods was high (92.9%), indicating that the animals were generally detected during these periods; however, only 27.7% of manually annotated pacing-like lion-time was retained as pacing-like by the complete pipeline, while a substantial proportion was classified as other active behavior. The automated measure should therefore not be interpreted as a complete estimate of true pacing prevalence. Rather, it identifies repeated back-and-forth movement meeting predefined geometric criteria and is best regarded as a screening indicator for periods warranting closer behavioral examination. The pacing results should therefore be regarded as exploratory indicators of stereotypic-like movement that can identify periods or individuals for closer investigation, rather than as definitive evidence of stereotypic behavior or poor welfare.

### 4.6. Methodological Considerations

A key strength of this study is the use of continuous video-based behavioral quantification across multiple enclosures. Traditional behavioral observation is often constrained by observer availability, restricted sampling periods and the difficulty of monitoring animals continuously over long timeframes [39]. The machine-learning approach enabled a large volume of video material to be converted into behavioral data and summarized across hours and days. This provides a useful framework for welfare-oriented monitoring in zoological settings, particularly when the aim is to identify broad behavioral patterns occurring outside conventional observation periods. Automated analysis may therefore function as a bridge between detailed manual ethological observations and large-scale continuous welfare monitoring [31,44,45,46].

The usefulness of this approach lies not only in reducing manual workload but also in allowing the same behavioral dataset to be examined from several complementary perspectives. From the classified output, it was possible to calculate time budgets, activity proportions, behavioral diversity, temporal instability, social interaction and pacing-related movement. This illustrates the potential of machine-learning-based behavioral analysis for descriptive and hypothesis-generating welfare research. Such approaches may be particularly valuable in zoo environments, where animals are monitored across complex enclosures, variable lighting conditions and extended periods. Continuous analysis can reveal patterns that may be difficult to identify from short manual observation sessions alone, including night-time behavior, temporal variation and relatively uncommon behavioral events [31,47,48,49].

However, the high temporal resolution of automated data does not overcome limitations in biological replication. Although thousands of behavioral observations can be generated from continuous footage, enclosure-level questions still require replication at the enclosure level. In the present study, the three enclosures provided detailed case comparisons but were insufficient for robust estimation of independent effects of space, complexity or social composition. Future studies designed to test such relationships would require substantially more independently sampled enclosures. Repeated temporal measurements could then be incorporated within hierarchical models while enclosure-level replication provides the information required for between-enclosure inference. The approach also remains dependent on the accuracy of behavioral classification and tracking. Some behaviors are easier to distinguish visually than others, and visually similar behaviors may be confused. This is particularly relevant under very dim or very bright recording conditions, where static objects or background features may occasionally be falsely detected as animals. In the spatial heatmaps, such errors may appear as highly concentrated hotspots representing repeated detections at the same location rather than true animal presence. More advanced camera systems and improved model performance may partially mitigate these limitations [47,49], but automated behavioral data should still be interpreted alongside model validation, data-quality assessment and biological knowledge. Prediction coverage also differed among institutions in the temporally held-out manual validation, with 59.8% of manually observed lion-time receiving a retained pipeline prediction in AAL, 56.2% in KBH and 33.8% in ODE [34]. Thus, although the total retained classified behavior time in the present analysis was similar among enclosures, the reported time budgets describe the distribution of behavior within retained classified output rather than complete coverage of all observable lion behavior. If detection or prediction failure is behaviorally non-random, differential coverage may influence the estimated behavioral proportions, and between-enclosure comparisons should therefore be interpreted with this limitation in mind.

The static night-time filter represents an additional source of uncertainty. Although it was designed to remove repeated detections of stationary background objects, a genuinely inactive lion could in principle exhibit sufficiently little centroid movement to meet the filtering criteria. The underlying assumption of the filter was, however, that the degree and persistence of near-zero movement required by the thresholds would be unlikely for a genuine lion detection, including an inactive animal, and would more commonly represent a static false detection. Nevertheless, occasional removal of genuine inactive observations cannot be excluded. Such errors would preferentially reduce the estimated inactive proportion and correspondingly increase the relative proportion of active behavior among retained predictions. Removal rates among filter-eligible windows were similar across enclosures, but this does not eliminate the possibility of behavior-specific filtering bias or differences in recording conditions among cameras and institutions.

The relatively short sampling period also limits the temporal representativeness of the behavioral time budgets. Data were collected over ten days in late December, spanning the Christmas and New Year period and a season with limited daylight. Behavioral proportions may therefore reflect winter-specific conditions or temporary changes in management and visitor patterns rather than longer-term annual activity. Rare behaviors are particularly sensitive to this limitation. For example, feeding behavior may be strongly influenced by the small number and timing of feeding events occurring within the ten-day window, especially where carcass feeding followed weekly schedules. Accordingly, low-frequency behavioral proportions should be interpreted as descriptive of the sampled period rather than as stable long-term behavioral rates.

Future studies would benefit from combining a larger number of enclosures with repeated sampling across seasons and more detailed measurements of enrichment, visitor presence, weather, feeding events and social stability. This would make it possible to distinguish more effectively between enclosure structure, management routines and social composition. Longitudinal monitoring within the same enclosure could also be particularly informative when conditions change, for example following alterations in group composition, enrichment or enclosure design, because animals can then be compared against their own previous behavioral patterns. Overall, the present study demonstrates both the potential and the limitations of machine-learning-based behavioral monitoring in zoo animal welfare research. With continued validation, careful quality control and greater biological replication, the approach could provide a useful tool for identifying welfare-relevant behavioral changes and supporting husbandry decision-making.

## 5. Conclusions

This study demonstrates the use of machine-learning-based video analysis for characterizing welfare-relevant behavioral patterns in captive lions across three contrasting zoo enclosures. The enclosures showed distinct behavioral profiles, but the observed differences did not follow simple gradients in their physical or social characteristics. Activity was highest in AAL, but did not increase consistently with either available space or structural complexity across the three enclosures. Behavioral diversity was lowest in AAL and highest for Shannon diversity in ODE, while temporal instability showed a different pattern, demonstrating that behavioral diversity and temporal stability capture distinct aspects of behavioral organization.

Social interaction did not increase consistently with pride size or cub presence. Pacing-like movement was concentrated in AAL while remaining very limited in KBH and ODE; however, because only 27.7% of manually annotated pacing-like lion-time was classified as pacing-like by the complete pipeline, this result should be treated as a screening signal for targeted behavioral examination rather than an estimate of true pacing prevalence or standalone evidence of poor welfare. Interpretation of the AAL profile is additionally limited by the euthanasia of the pride’s only adult male five days before data collection, and the observed behavioral pattern may therefore partly reflect this recent social disruption. These patterns illustrate that lion behavior is likely shaped by multiple interacting characteristics of the animals, their social groups, enclosure design, management history and husbandry routines. With only three independent enclosures, the contribution of these factors cannot be separated, and the findings should therefore be interpreted as exploratory case comparisons rather than estimates of causal enclosure effects.

Overall, the study supports automated behavioral monitoring as a promising tool for zoo welfare research. Continuous machine-learning-based analysis can generate detailed information on behavioral time budgets, diversity, temporal variation, social behavior, spatial use and pacing-like movement that would be difficult to obtain through conventional observation alone. When combined with appropriate biological replication, careful model validation and contextual knowledge from animal care staff, these methods may contribute to more systematic and evidence-based monitoring of captive animal welfare.

## Figures and Tables

**Figure 1:**
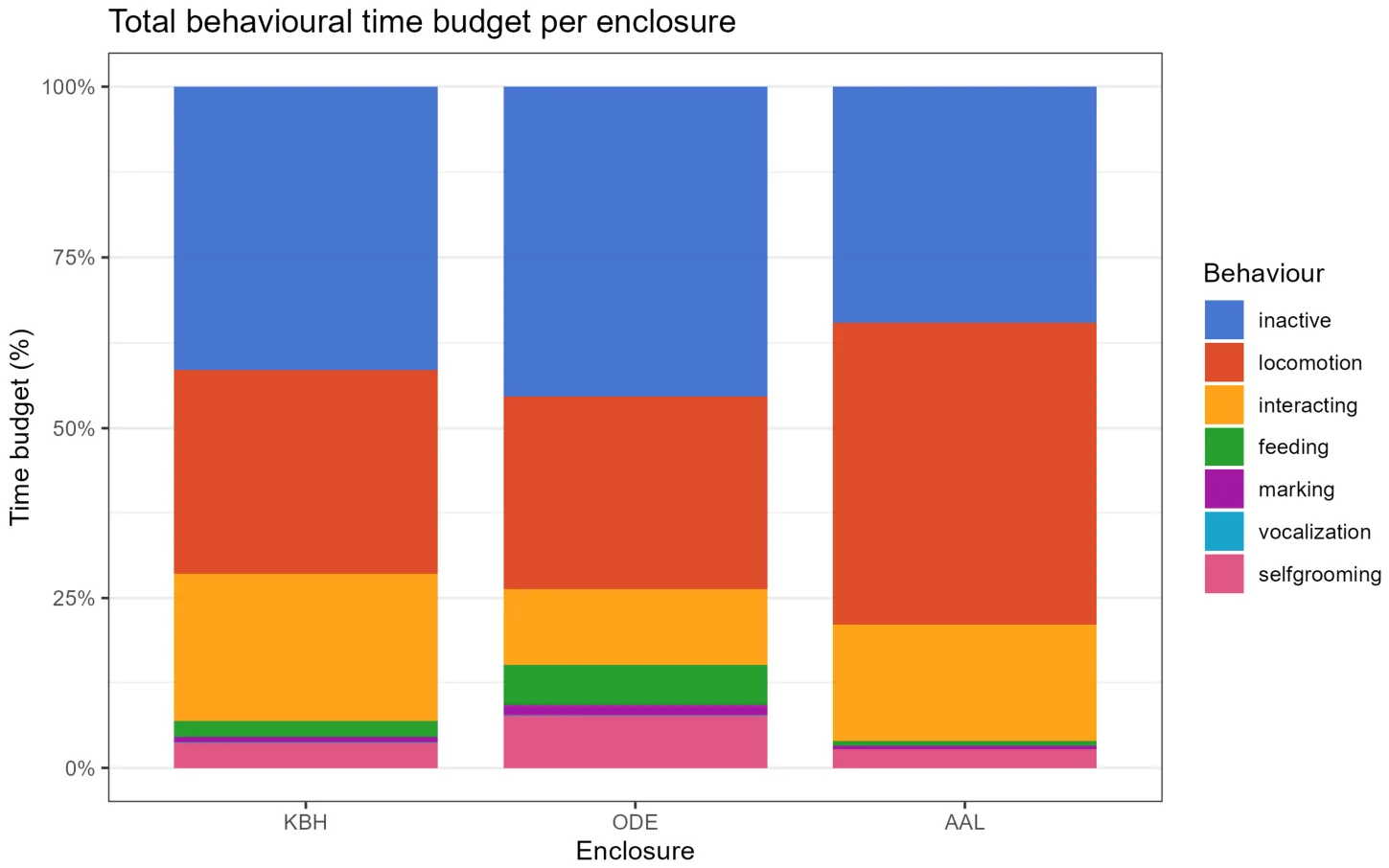
Total time budget displaying overall enclosure-level behavior across all lion types. Behavior is noted as proportion of behavior of total observed time.

**Figure 2:**
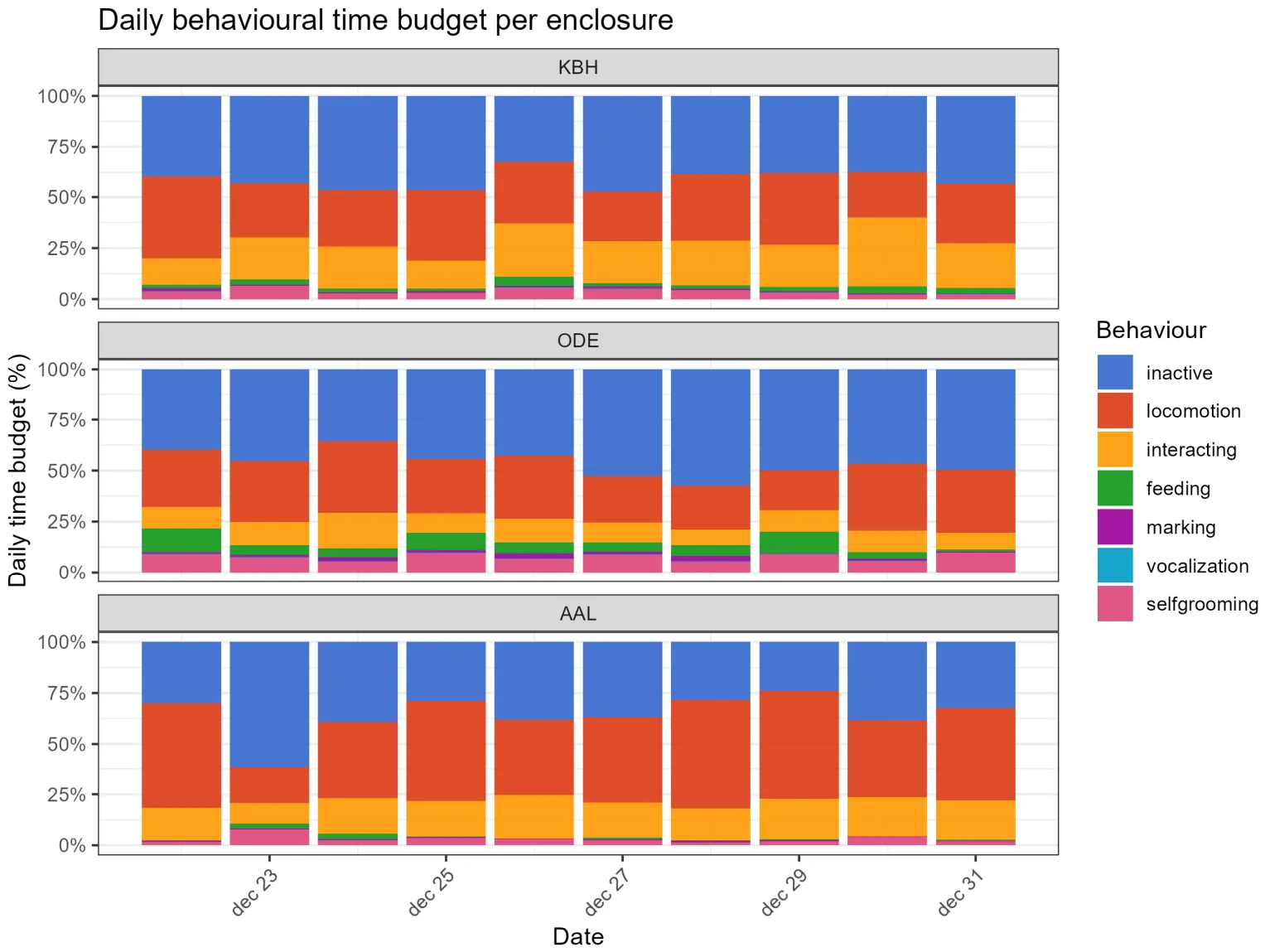
Time budget displaying daily enclosure-level behavior of all lion types. Behavior is noted as proportion of behavior of total observed time.

**Figure 3:**
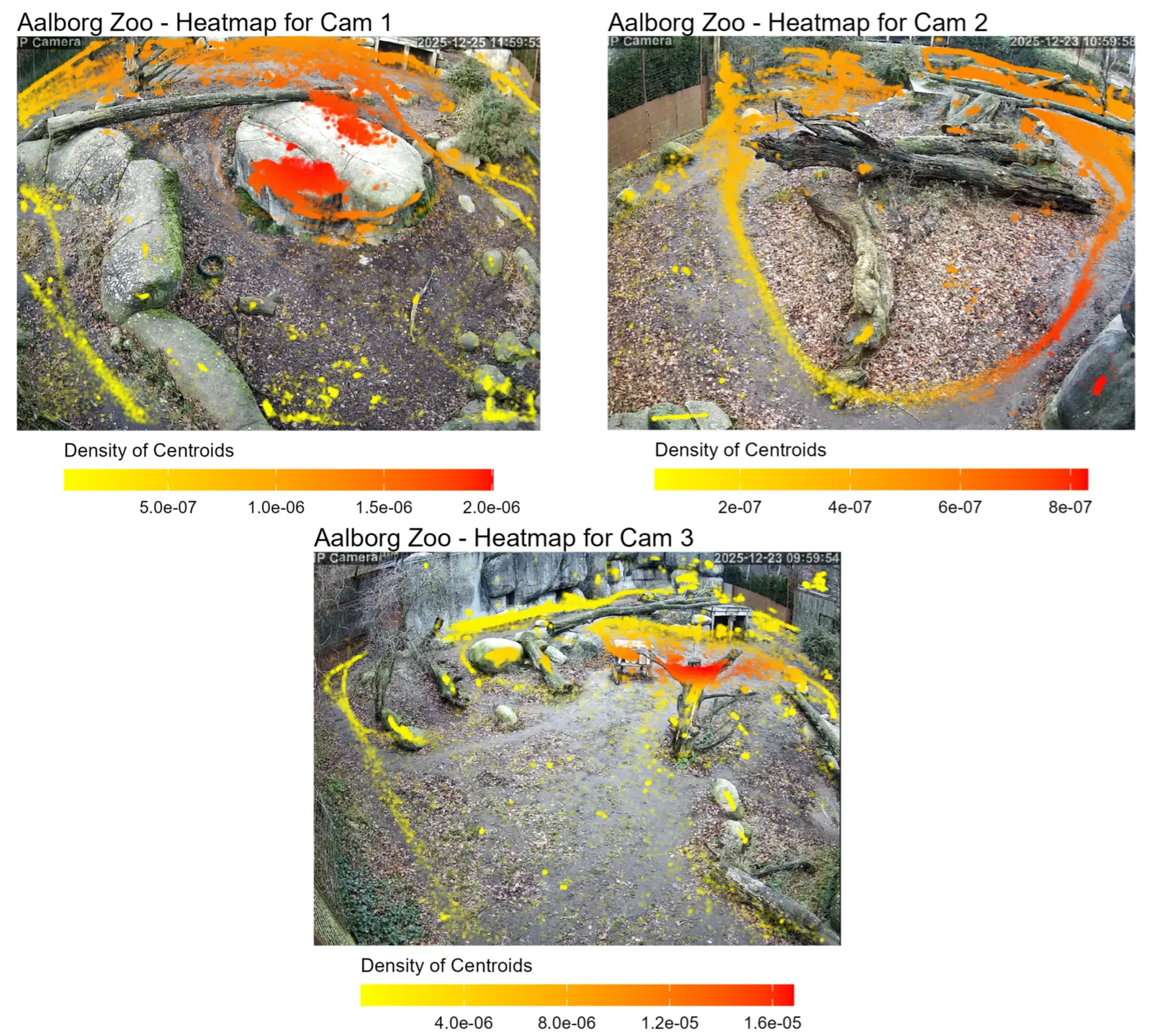
Spatial heatmaps of lion centroid positions generated from automated video tracking in the Aalborg Zoo enclosure. Density estimates were calculated from the centroid coordinates of detected individuals across the study period. Warmer colors (orange-red) indicate areas with higher density, while cooler colors (yellow) represent less frequently used locations. The three panels show the spatial distribution of lion activity from Camera 1, Camera 2, and Camera 3, respectively, showing areas with higher densities of detected centroid positions within each camera view.

**Figure 4:**
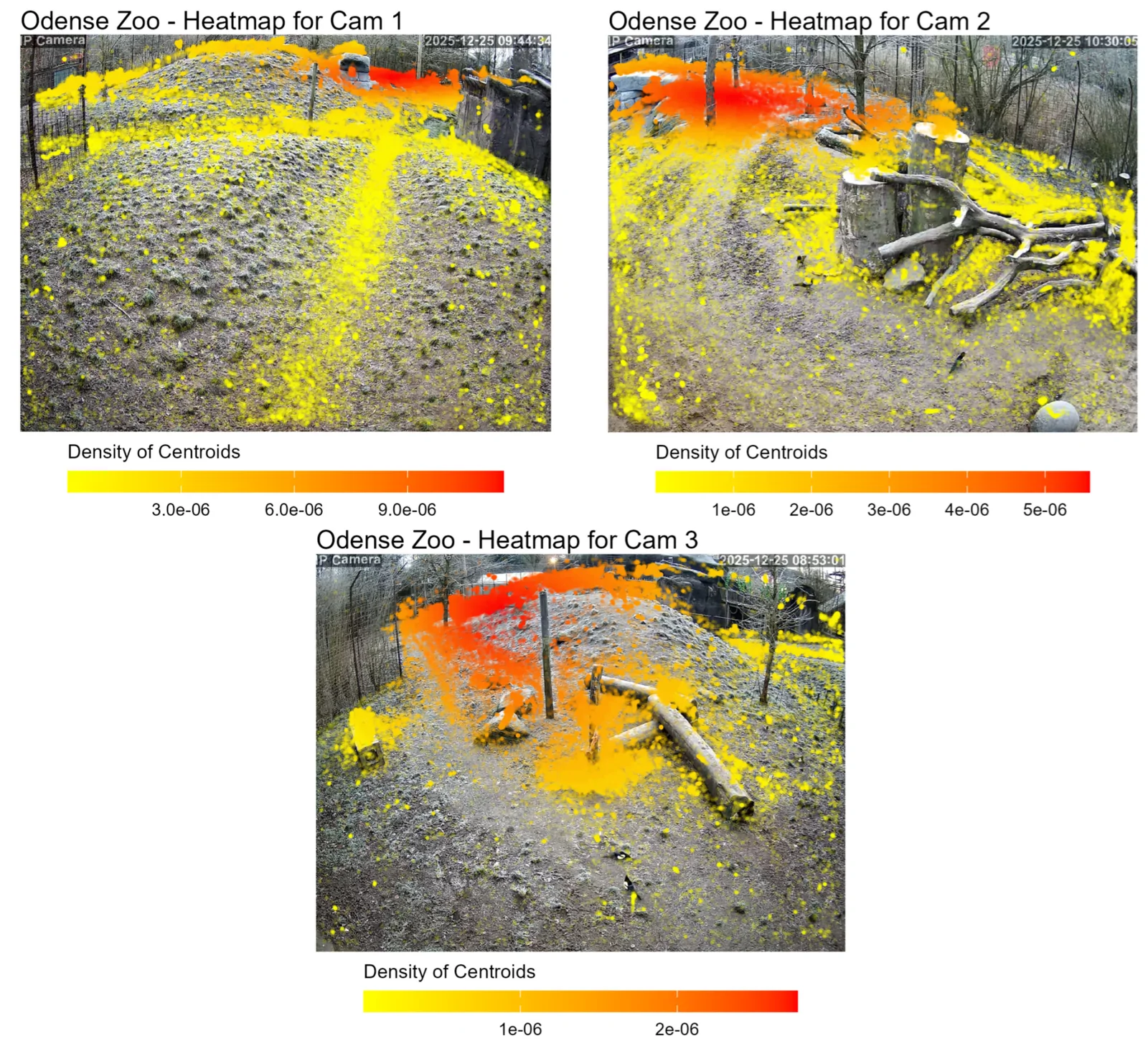
Spatial heatmaps of lion centroid positions generated from automated video tracking in the Odense Zoo enclosure. Density estimates were calculated from the centroid coordinates of detected individuals across the study period. Warmer colors (orange-red) indicate areas with higher density, while cooler colors (yellow) represent less frequently used locations. The three panels show the spatial distribution of lion activity from Camera 1, Camera 2, and Camera 3, respectively, showing areas with higher densities of detected centroid positions within each camera view.

**Figure 5:**
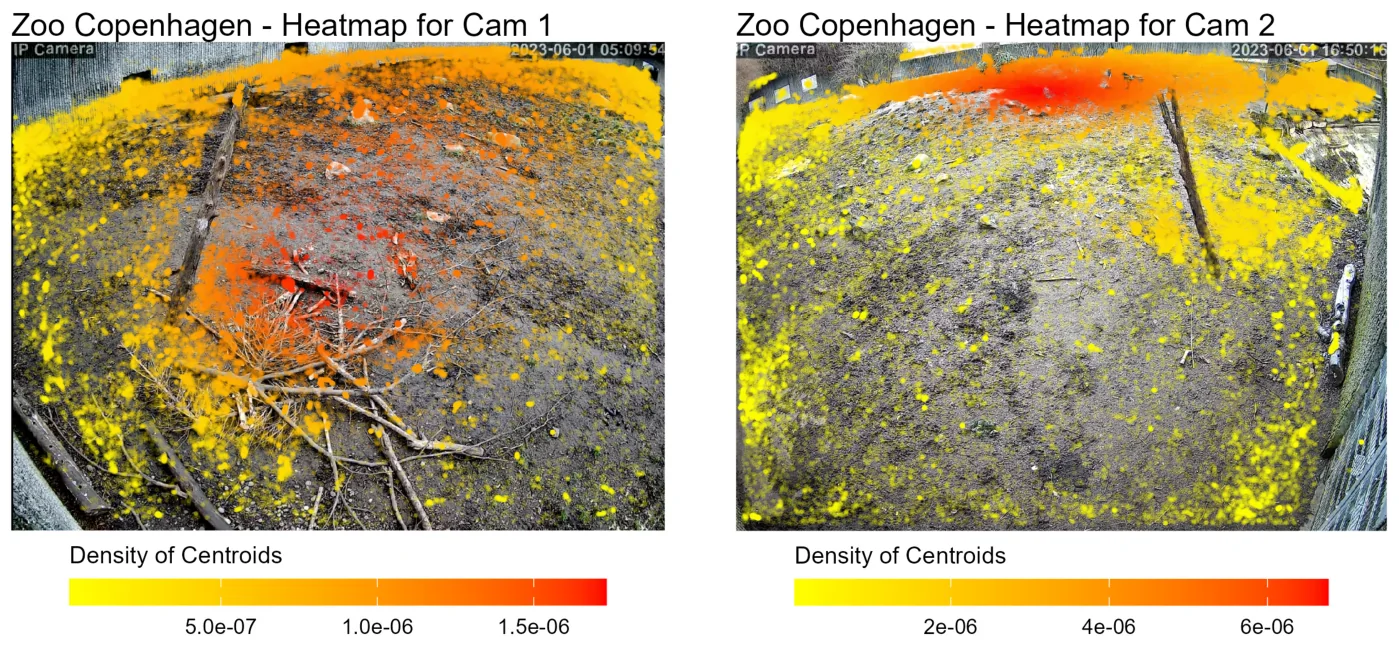
Spatial heatmaps of lion centroid positions generated from automated video tracking in the Zoo Copenhagen enclosure. Density estimates were calculated from the centroid coordinates of detected individuals across the study period. Warmer colors (orange-red) indicate areas with higher density, while cooler colors (yellow) represent less frequently used locations. The two panels show the spatial distribution of lion activity from Camera 1 and Camera 2, respectively, showing areas with higher densities of detected centroid positions within each camera view.

**Table 1:** Fact sheet for Aalborg Zoo.

Category	Description
Enclosure type	Mixed indoor–outdoor exhibit
Enclosure size	700 m^2^
Space per lion	350 m^2^
Access to indoor/outdoor areas	Lions have free access to both indoor and outdoor areas throughout the year, except during periods of enclosure maintenance and cleaning.
Layout and design	The enclosure consisted of a naturalistic exhibit with varied terrain, elevation changes, vegetation (bushes and trees), multiple substrate types, such as rocks, grass and dirt. Feeding and resting areas were distributed throughout the enclosure, which also included a sheltered area in the form of a shed.
Indoor facilities	Indoor areas were accessible year-round and remained visible to the public.
Pride structure	2 adult females (mixed group).
Daily routines	Cleaning approximately once per week, often coordinated with feeding.
Enrichment	Scheduled enrichment every Sunday, which includes olfactory enrichment, feeding enrichment and structural elements.
Feeding routines	Main feeding once per week (Tuesday), primarily carcass.
Complexity score	2

**Table 2:** Fact sheet for Zoo Copenhagen.

Category	Description
Enclosure type	Outdoor exhibit with an indoor area that was not accessible to visitors.
Enclosure size	703 * m^2^
Space per lion	100.4 m^2^
Access to indoor/outdoor areas	Lions were temporarily housed indoors during morning cleaning of the outdoor enclosure and, on occasion, during indoor cleaning later in the day
Layout and design	The enclosure consisted of a naturalistic exhibit with varied terrain, elevation changes, vegetation (bushes and trees), and multiple substrate types, including rocks, grass and dirt. Feeding and resting areas were distributed throughout the enclosure, which also included a sheltered area in the form of a cave and a heated rock. The outdoor exhibit was surrounded by a moat that served as a natural barrier between the lions and visitors.
Indoor facilities	Indoor areas were accessible throughout the year and divided into five holding boxes.
Pride structure	1 adult male, 2 adult females (mixed group) and 4 belonging to the mixed group.
Daily routines	The outdoor enclosure was cleaned each morning before opening to the public, while the indoor area was cleaned later in the day.
Enrichment	No fixed enrichment schedule was followed; instead, enrichment varied daily and included feeding-related, olfactory, and tactile stimuli.
Feeding routines	Irregular feeding times; combination of scatter feeding and carcass feeding.
Complexity score	1

* Not including the moat surrounding the outdoor area.

**Table 3:** Fact sheet for Odense Zoo.

Category	Description
Enclosure type	Outdoor exhibit with some indoor area accessible to visitors.
Enclosure size	Approximately 1200–1300 m^2^
Space per lion	138.9 m^2^
Access to indoor/outdoor areas	Lions have free access to both indoor and outdoor areas throughout the year, except during periods of enclosure, maintenance and cleaning.
Layout and design	The enclosure consisted of a naturalistic exhibit with varied terrain, hill, vegetation (bushes and trees), and multiple substrate types, including rocks, grass and dirt, and a pond. Feeding and resting areas were distributed throughout the enclosure, which also included a sheltered area in the form of a cave and a heated rock.
Indoor facilities	Indoor areas were accessible throughout the year.
Pride structure	1 adult male, 3 adult females (mixed group), 2 belonging to the mixed group and 3 cubs.
Daily routines	Weekly cleaning of indoor area and outdoor cleaning as needed.
Enrichment	2–3 times per week which included cognitive, sensory, physical and feeding enrichment.
Feeding routines	Fixed feeding once per week (Wednesday at 12:00), primarily carcass feeding outdoors.
Complexity score	3

**Table 4:** Ethogram of selected behaviors to use for LabGym Categorizer. * Categories requiring additional post-processing and not treated as direct categorizer outputs. ** Sex-specific category applied only to adult males.

Behavior	Description
Feeding	The lion engages in feeding-related behavior, including chewing or tearing meat, manipulating food or objects with paws or jaws, and carrying or dragging food items. Carcass handling, such as dragging, covering or repositioning remains, is also included. Drinking and foraging-related behaviors, such as sniffing or searching around potential food sources, are included in this category.
Inactive	The lion remains stationary or nearly stationary, either sitting, lying or standing, without meeting the criteria for another specific behavior. This category includes resting or sleeping, vigilance or observing the environment, stretching and yawning. The model does not distinguish among these low-movement states.
Interacting	The lion engages in social interaction with conspecifics. This includes non-aggressive behaviors such as sniffing, rubbing, approaching, lying in close proximity, social grooming and play, as well as reproductive, aggressive or agonistic interactions such as courtship, mounting, mock fighting, chasing, swatting, biting or fighting.
Locomotion	The lion moves from one location to another by walking, trotting, running, jumping or climbing, without engaging in another distinct behavior.
Marking	The lion deposits scent or other signals by rubbing, urinating, defecating or clawing against trees or other surfaces.
Pacing *	The lion moves systematically back and forth over an extended period of time.
Self-Grooming	The lion licks, scratches or rubs its own body to clean or maintain its fur or claws.
Vocalization **	The lion roars to communicate with conspecifics or signal its presence.

**Figure A1:**
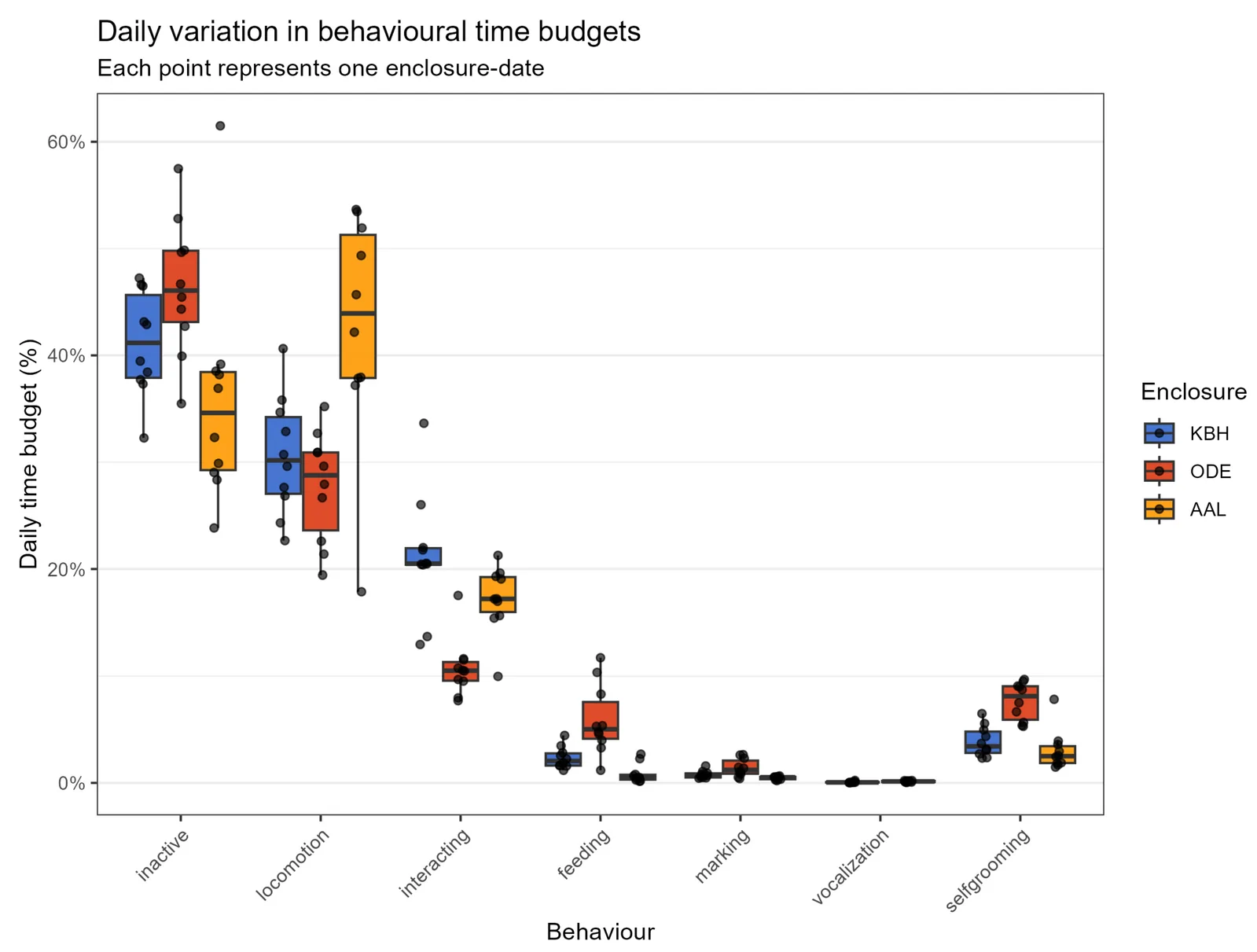
Daily variation in behavioral time budgets across enclosures. Boxplots show the distribution of daily percentages for each behavior within each enclosure. Each point represents one enclosure-day. Colors indicate enclosure. This figure illustrates how the relative time allocated to each behavior varied across recording days and allows comparison of day-to-day behavioral variation between enclosures.

**Figure A2:**
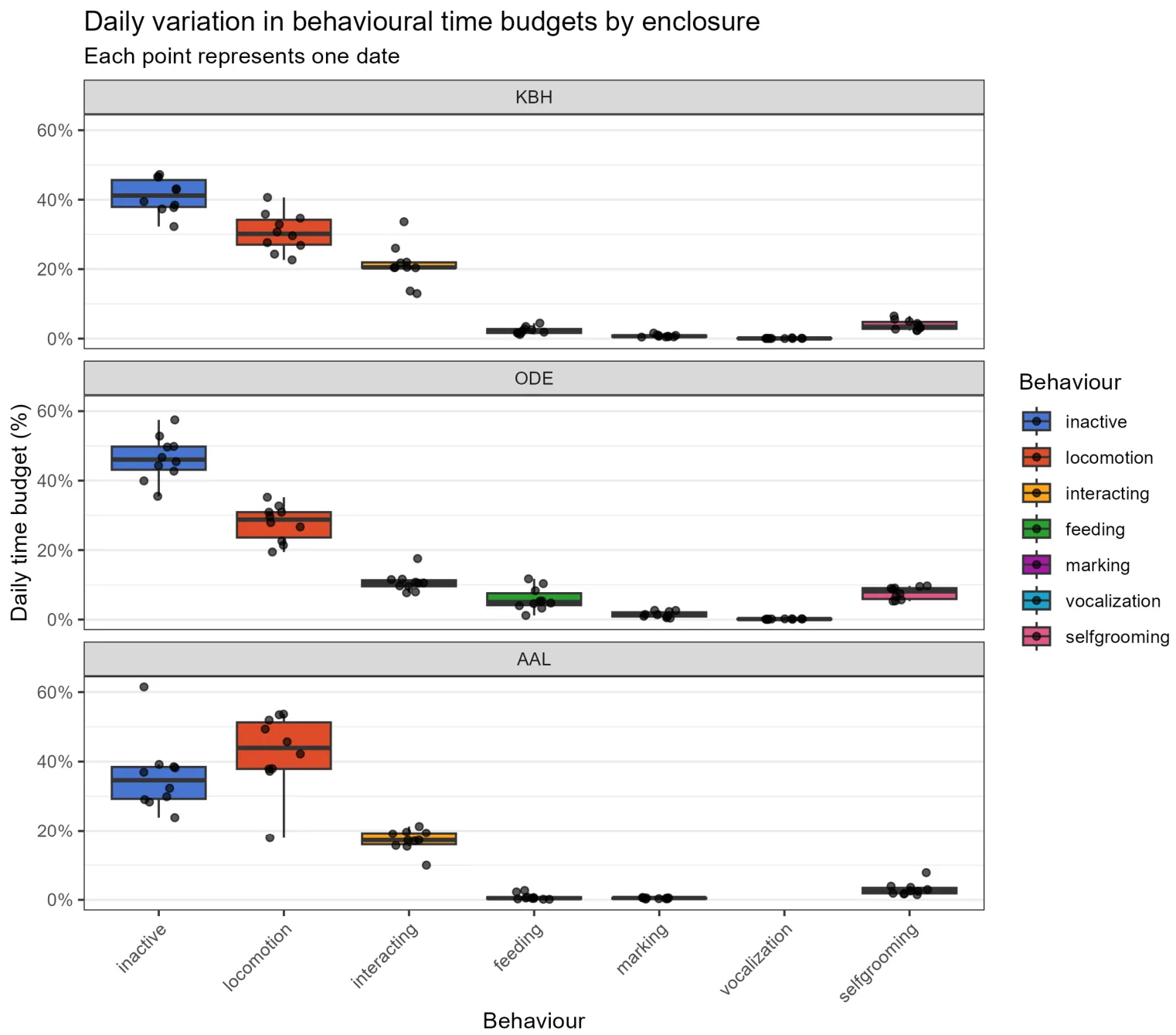
Daily variation in behavioral time budgets within each enclosure. Boxplots show the distribution of daily percentages for each behavior across recording days, with separate panels for each enclosure. Each point represents one enclosure-day. This figure provides a within-enclosure view of behavioral variation and shows whether behavioral composition was consistent or variable across the study period.

## Data Availability

The data presented in this study are available on request from the corresponding authors.

## Appendix A

### Lion Taxonomy

Lions belong to the Felidae family and the genus *Panthera*. The classification of lion sub-species has been debated and revised several times. According to the IUCN and supporting literature, two subspecies are currently recognized: *Panthera leo leo* and *Panthera leo melanochaita* [20,50,51]. The Asian lion (*Panthera leo persica*) is no longer considered a separate subspecies, but rather a haplogroup within *P. l. leo*, due to its low nucleotide divergence from that subspecies [20,50,51]. Both *P. l. leo* and *P. l. melanochaita* are often referred to as African lions. *P. l. leo* are distributed in Central and West Africa as well as in Asia, while *P. l. melanochaita* are found in Southern and East Africa. There is a notable overlap between the two subspecies distribution in Ethiopia and recent studies identified significant genetic overlap in these areas. This is a natural occurrence of hybridization and no management intervention is planned [20]. Although differences in social structure have been described in certain populations, such as smaller and more sexually segregated groups among Asiatic lions compared to larger, mixed-sex prides in parts of Africa. These variations are understood to be shaped primarily by environmental context, such as resource availability, rather than fixed sub-species traits [26,27]. Available research on captive lions generally focuses on a single population, making it difficult to assess whether any observed behaviors are subspecies-specific or context-dependent. However, existing husbandry guidelines, such as those outlined in the AZA (Association of Zoos and Aquariums) Lion (*Panthera leo*) Care Manual [41], do not differentiate between subspecies and recommend comparable management practices for all lions in zoological settings. Due to natural hybridization in the wild, hybridization occurring in zoological settings, and the ongoing uncertainty surrounding subspecies boundaries, as well as similar management in captivity, this study does not distinguish between lion subspecies.

## Appendix B

**Table A1:** Internal development-validation performance of the LabGym behavior categorizer. These results describe the internal categorizer validation and should be distinguished from the temporally held-out applied validation of the complete analysis pipeline summarized in Table A2 and Table A3.

Behavior	Precision	Recall	F1-Score	Support
Feeding	0.63	0.59	0.61	32
Inactive	0.61	0.62	0.62	53
Interacting	0.71	0.58	0.64	71
Locomotion	0.58	0.75	0.66	51
Marking	0.33	0.25	0.29	12
Self-grooming	0.73	0.58	0.65	19
Vocalization	0.59	0.76	0.67	25
Accuracy			0.62	263
Macro average	0.60	0.59	0.59	263
Weighted average	0.63	0.62	0.62	263

Behavioral predictions were retained only when confidence was ≥0.60. This threshold was established during model development, before evaluation of the temporally held-out validation footage, as a balance between retaining potentially correct predictions and excluding low-confidence classifications.

**Table A2:** Prediction coverage and end-to-end versus conditional accuracy in the temporally held-out applied validation. Conditional accuracy includes only manually annotated lion-time with a retained pipeline prediction.

Scope	Prediction Coverage	Fine End-to-End Accuracy	Fine Conditional Accuracy	Grouped End-to-End Accuracy	Grouped Conditional Accuracy
Overall	52.6%	0.231	0.440	0.317	0.602
AAL	59.8%	0.250	0.418	0.290	0.484
KBH	56.2%	0.253	0.451	0.390	0.695
ODE	33.8%	0.160	0.472	0.226	0.668

**Table A3:** Behavior-specific performance in the temporally held-out applied validation. Prediction coverage is the proportion of manually annotated lion-time receiving a retained pipeline prediction. Correct classification from total manual lion-time includes uncovered time in the denominator.

Manual Behavior	Prediction Coverage	Correctly Classified from Total Manual Lion-Time
Feeding	51.4%	6.7%
Inactive	45.7%	21.7%
Interacting	37.6%	20.0%
Locomotion	52.5%	42.0%
Marking	52.3%	3.9%
Self-grooming	40.8%	4.0%
Pacing-like locomotion	92.9%	27.7%
